# Emerging insights into the roles of ANGPTL8 beyond glucose and lipid metabolism

**DOI:** 10.3389/fphys.2023.1275485

**Published:** 2023-12-01

**Authors:** Huimin Ye, Qunchuan Zong, Huajie Zou, Ruixia Zhang

**Affiliations:** ^1^ Department of Endocrinology and Metabolism, The Affiliated Hospital of Qinghai University, Xining, China; ^2^ Department of Traumatology and Orthopaedics, The Affiliated Hospital of Qinghai University, Xining, China

**Keywords:** ANGPTL8, inflammation, tumour, circulatory system, ectopic lipid deposition

## Abstract

Angiopoietin-like protein 8 (ANGPTL8) is a secreted protein predominantly expressed in liver and adipose tissue. ANGPTL8 modulates the clearance of triglycerides (TGs) by suppressing the activity of lipoprotein lipase (LPL) within the plasma. Previous studies found that circulating ANGPTL8 levels were significantly increased in metabolic disorder-related diseases, such as type 2 diabetes mellitus (T2DM), obesity, metabolic syndrome and nonalcoholic fatty liver disease (NAFLD). Whether ANGPTL8 has a direct pathogenic role in these diseases remains to be determined. In this review, we summarize the emerging roles of ANGPTL8 in the regulation of inflammation, tumours, circulatory system-related diseases, and ectopic lipid deposition, which may provide new insights into the diverse functions of ANGPTL8 in various diseases beyond its well-established functions in glucose and lipid metabolism.

## 1 Introduction

Angiopoietin-like protein 8 (ANGPTL8), also known as betatrophin, TD26, and lipasin, is released from the liver and adipose tissue (Guo et al., 2021). ANGPTL8 is a novel but atypical member of the ANGPTL family, as it lacks the typical fibrinogen-like domain. However, its N-terminal domain shares homology with ANGPTL3 and ANGPTL4 (Zhang, 2016; Guo et al., 2021). Previous studies have demonstrated the role of the ANGPTL3-4-8 model in inhibiting the activity of lipoprotein lipase (LPL), which is a rate-limiting enzyme for triglyceride (TG) hydrolysis and plasma TG clearance (Zhang, 2012; Zhang, 2016). Feeding induces ANGPTL8 expression and inhibits ANGPTL4 expression. Then, the ANGPTL8–ANGPTL3 pathway is activated and inhibits LPL in cardiac and skeletal muscles, allowing circulating TG to be taken up by white adipose tissue (WAT) to store energy. Conversely, ANGPTL8 levels are reduced during fasting, while ANGPTL4 levels are increased. Thus, TGs are directed towards the muscles to provide energy (Zhang, 2012; Zhang, 2016). The expression of ANGPTL8 is regulated by nutrition (Zhang, 2012), insulin (Lu et al., 2017) and thyroid hormone (Tseng et al., 2014). ANGPTL8 is closely associated with metabolic disorder-related diseases, such as type 2 diabetes mellitus (T2DM) (Zou et al., 2020a; Zou et al., 2020b), nonalcoholic fatty liver disease (NAFLD) (von Loeffelholz et al., 2017), obesity and metabolic syndrome (Mele et al., 2017; Wang et al., 2016). However, the precise roles of ANGPTL8 in these diseases are not known (Abu-Farha et al., 2020). Whether ANGPTL8 is involved in other pathophysiological processes beyond glucose and lipid metabolism remains to be explored. With the deepening of research, evolving implications of ANGPTL8 actions in the regulation of inflammation, tumours, circulatory system-related diseases, and ectopic lipid deposition have been reported. Therefore, in this work we review recent findings related to ANGPTL8 and aim to provide new insights into the diverse functions of ANGPTL8 in various diseases.

## 2 ANGPTL8 and inflammation

**FIGURE 1:**
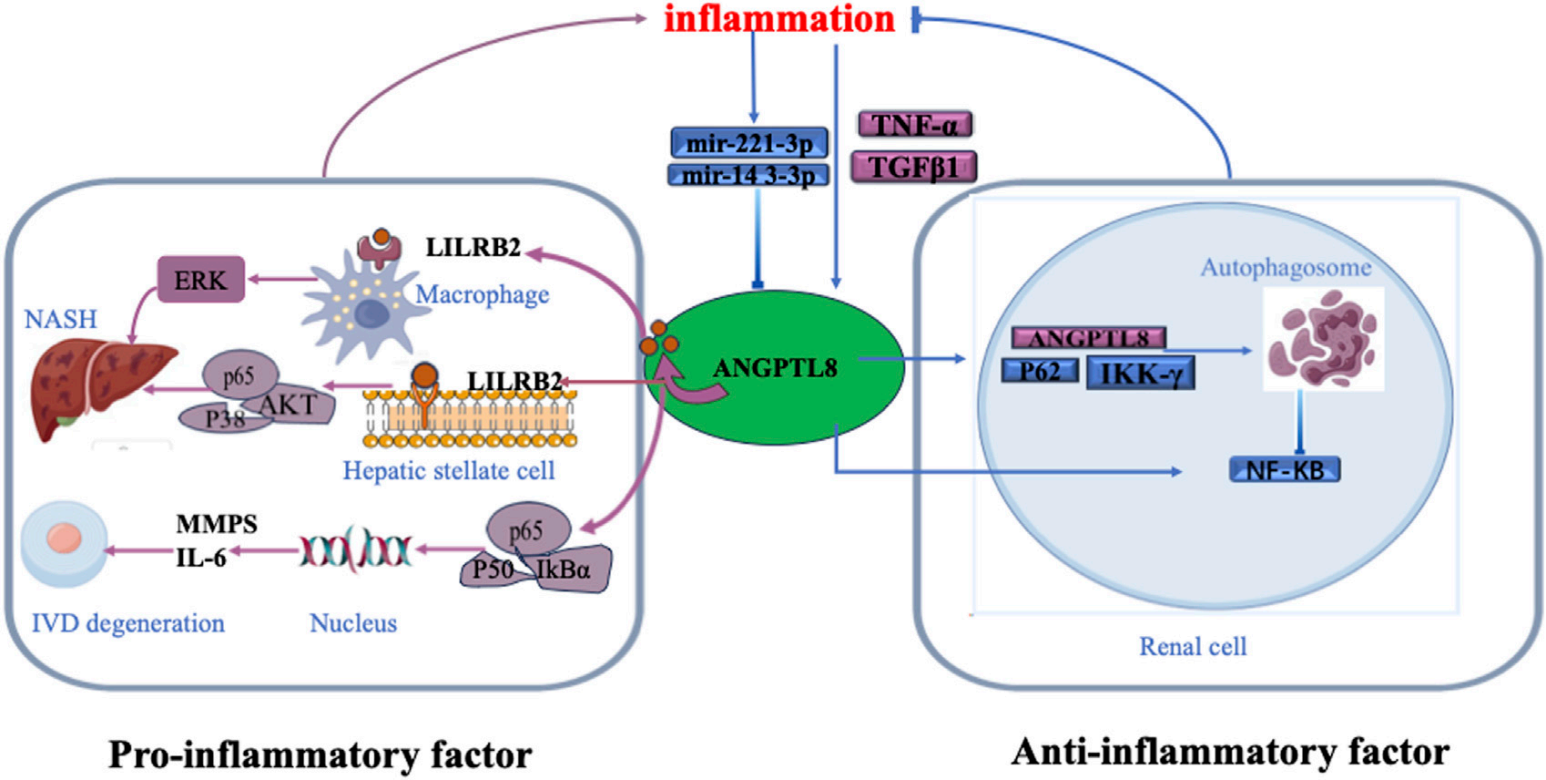
The role of ANGPTL8 in inflammation. Bladder urothelial carcinoma (BLCA), breast invasive carcinoma (BRCA), clear cell renal cell carcinoma (ccRcc), cholangiocarcinoma (CHOL), colon adenocarcinoma (COAD), glioblastoma (GBM), hepatocellular carcinoma (HCC), kidney chromophobe (KICH), kidney renal clear cell carcinoma (KIRC), lung adenocarcinoma (LUAD), lung squamous cell carcinoma (LUSC), pancreatic adenocarcinoma (PAAD), pheochromocytoma and paraganglioma (PCPG), rectum adenocarcinoma (READ, sarcoma (SARC), stomach adenocarcinoma (STAD), thymoma (THYM), uterine corpus endometrial carcinoma (UCEC), uterine carcinosarcoma (UCS), and uveal melanoma (UVM).

Previous studies have found that circulating ANGPTL8 levels are elevated in inflammation-related diseases, such as systemic inflammatory response syndrome (SIRS) (Zhang et al., 2017), T2DM (Chen et al., 2015), atherosclerosis (Jiao et al., 2018), and NAFLD (Vatner et al., 2018), and are associated with disease severity. However, the precise roles of ANGPTL8 in these diseases are not known. Zhang et al. (2017) found an increase in ANGPTL8 levels in patients with severe sepsis. Furthermore, they observed a notable connection between circulating ANGPTL8 levels and the acute inflammatory response induced by lipopolysaccharide (LPS) in mice (Zhang et al., 2017). Liao et al. (2019) demonstrated the detrimental role of ANGPTL8 expression in the pathogenesis of intervertebral disc degeneration (IDD), and knockdown of ANGPTL8 ameliorated IDD progression. (Figure 1) (Liao et al., 2019). They found that tumour necrosis factor-α (TNF-α) stimulated the expression of ANGPTL8 in bone marrow cells. Then, ANGPTL8 increased the production of matrix metalloproteinases (MMPs) and inflammatory cytokines while reducing the level of type II collagen in bone marrow cells through inhibition of NF-κB signalling activation. A previous study showed that fatty acids (FAs) upregulate ANGPTL8 expression within the liver, consequently triggering the expression and secretion of transforming growth factor-beta1 (TGFβ1) (Zhang et al., 2023). Subsequently, secreted TGFβ1 further stimulates ANGPTL8 expression. ANGPTL8 combines with the leukocyte immunoglobulin-like receptor B (LILRB2) receptor and then acts as a proinflammatory factor that stimulates hepatic stellate cells (HSCs). This then induces extracellular signal-regulated kinase (ERK) signalling and upregulates genes associated with liver fibrosis (Figure 1) (Zhang et al., 2023). However, another study demonstrated that ANGPTL8 bound with paired immunoglobulin-like receptor B (PirB)/LILRB2 on macrophages, but not HSCs, during nonalcoholic steatohepatitis (NASH) (Li et al., 2023). This PirB/LILRB2-ANGPTL8 interaction facilitated the conversion of hepatic macrophages into a proinflammatory phenotype by intensifying phosphorylation signals of P38, protein kinase B (AKT), and P65, thereby exacerbating hepatocyte lipid accumulation and propelling the progression from simple hepatic steatosis to steatohepatitis (Figure 1) (Li et al., 2023). Based on the above findings, ANGPTL8 may act as a proinflammatory factor.

However, several other studies have suggested that ANGPTL8 may be a negative regulator of inflammation. ANGPTL8 was reported to exert a negative regulatory influence on nuclear factor kappa-B (NF-κB) by facilitating the autophagic degradation of inhibitory nuclear factor kinase κB-γ (IKKγ) within the cellular context (Zhang et al., 2017). Notably, NF-κB functions as a pivotal transcription factor within the intricate framework of inflammatory signalling (Zhang et al., 2017). Moreover, the well-established inducer of NF-κB activation, TNF-α, was observed to elevate ANGPTL8 expression in human hepatocytes (HepG2). ANGPTL8, through its interaction with sequestosome1 (p62) and autophagy receptors, facilitates targeted autophagic breakdown of IKKγ, thereby resulting in dampened NF-κB activity (Figure 1) (Zhang et al., 2017; Zhang et al., 2018). MicroRNAs (miRNAs) are small noncoding RNA molecules that regulate gene expression and have emerged as important components in the regulation of inflammation. MiR-221-3p is upregulated under inflammatory conditions, but there is a negative correlation between miR-221-3p in subcutaneous adipose tissue biopsy samples and plasma ANGPTL8 levels (Figure 1) (Mysore et al., 2017). Another miRNA, MiR-143-3p, has also been shown to be modulated by inflammation and downregulate ANGPTL8 in hepatocytes (Figure 1) (DiStefano, 2019). These studies indicated that ANGPTL8 may serve as a “brake” in certain inflammatory pathways. As shown in Figure 1, the dual roles of ANGPTL8 in inflammation suggest potential divergence in its intracellular function compared to its role as a secreted hormone in other physiological contexts, such as blood vessels (Abu-Farha et al., 2020).

## 3 ANGPTL8 and cancer

Increasing evidence suggests that ANGPTL8 is associated with cancer development and prognosis. However, ANGPLT8 levels and the association between ANGPTL8 and prognosis also differ among various cancers.

### 3.1 Liver cancer

ANGPTL8 is secreted by the liver and acts as an inflammatory factor in the pathophysiology of liver cancer. Initial investigations have established an association between increased serum concentrations of ANGPTL8 and NAFLD and its severity (von Loeffelholz et al., 2017; Lee et al., 2016; Cengiz et al., 2016). Furthermore, ANGPTL8 expression levels are also elevated in the livers of NAFLD mice (Lee et al., 2016), which is potentially attributable to its involvement in glucose and lipid metabolic pathways. NAFLD can further progress to liver fibrosis and liver cancer. Clinical investigations have identified elevated serum ANGPTL8 levels in individuals with liver fibrosis and liver cancer (Vatner et al., 2018; Pazgan-Simon et al., 2020; Wang et al., 2018). In the study by Wang et al. (2018), ANGPTL8 was highly expressed in hepatocellular carcinoma (HCC) tissues when compared to matched normal tissues, emerging as an independent predictor of overall survival and recurrence-free survival among HCC patients. ANGPTL8 significantly augments lipid synthesis within HCC cells and promotes tumour growth and metastasis by modulating the immune response within the tumour microenvironment; therefore, it was named “tumour-derived factor 26” (TD26) (Wang et al., 2018). Zhang et al. (2023) also found that the serum concentrations of ANGPTL8 in patients with liver cirrhosis and HCC increased by 1.57-fold and 2.14-fold, respectively. As discussed above, their findings indicated that ANGPTL8 expedites hepatic fibrosis through the LILRB2/ERK signalling pathways in HSCs. According to Gao et al. (2023), ANGPTL8 plays a dual role in hepatocarcinogenesis by promoting both tumour cell proliferation and immune evasion: 1) In hepatocytes, ANGPTL8-triggered LILRB2/PIRB activation regulates the ROS/ERK pathway, upregulating autophagy and promoting HCC cell growth; 2) In macrophages, the LILRB2/PIRB-ANGPTL8 interaction drives polarization towards the M2 phenotype, leading to suppression of the antitumour immune response within the tumour microenvironment, ultimately allowing tumorigenic HCC cells to evade immune surveillance (Gao et al., 2023). Nevertheless, Li et al. (2023) investigated PirB expression across various cell types in liver tissue (hepatocytes, HSCs, endothelial cells, and hepatic immunocytes) and determined that PirB is predominantly expressed in hepatic macrophages. Therefore, the effect of the PirB/LILRB2-ANGPTL8 interaction in different cell types in liver tissue remains to be further investigated.

However, another study suggested that the elevated expression of ANGPTL8 in HCC cells may inhibit cell proliferation by affecting the Wnt signalling pathway, upregulating the tumour suppressor gene WNT inhibitory factor-1 (WIF1) and downregulating β-catenin (Monzavi et al., 2019). In their opinion, ANGPTL8 may act as a moderate suppressor of human liver carcinoma cells.

### 3.2 Renal cell carcinoma

Our prospective study found that serum levels of ANGPTL8 are associated with renal dysfunction (Zou et al., 2021), consistent with other observational studies (Maurer et al., 2017). It has been suggested that ANGPTL8 may be an advanced endocrine regulatory factor involved in the development of diabetic kidney disease (Chen et al., 2016), which suggests that ANGPTL8 is closely associated with renal disease.

Renal cell carcinoma (RCC) is the most common type of kidney cancer, and clear cell renal cell carcinoma (ccRCC) constitutes approximately 70% of all renal cancer cases and correlates with chronic inflammation (Fox et al., 2013). ANGPTL8 gene expression was observed to be significantly increased in ccRCC tissues (Matsukawa et al., 2023). They found that ANGPTL8 was induced by inflammation and then sustained the generation of chemokines via the NF-κB signalling pathway (Matsukawa et al., 2023). Consequently, these chemokines attract immune suppressor cells to the tumour microenvironment and maintain the undifferentiated status of ccRCC cells. These results indicate that ANGPTL8 can promote tumour progression by establishing the tumour microenvironment. It was reported that patients with high ANGPTL8 expression were associated with a lower 5-year survival rate of kidney renal clear cell carcinoma (KIRC)/ccRCC (Xu et al., 2021). Based on the above, ANGPTL8 may serve as a novel unfavourable prognostic marker in renal cancer. However, the protein expression of ANGPTL8 displayed opposite results in ccRCC/KIRC samples from patients in different cancer stages, years old age groups and weight categories (Xu et al., 2021). The apparently contradictory results in gene and protein expression of ANGPTL8/betatrophin in ccRCC/KIRC must be further validated.

### 3.3 Other cancers

As shown in Table 1, analysis of various databases has shown significantly decreased expression of the ANGPTL8 gene in lung squamous cell carcinoma (LUSC), lung adenocarcinoma (LUAD), breast invasive carcinoma (BRCA), glioblastoma (GBM), cholangiocarcinoma (CHOL), uterine corpus endometrial carcinoma (UCEC), bladder urothelial carcinoma (BLCA), and kidney chromophobe (KICH) compared to their respective normal samples (Xu et al., 2021; Iqbal et al., 2023). Conversely, ANGPTL8 levels are significantly increased in HCC (Wang et al., 2018), KIRC/ccRCC (Matsukawa et al., 2023; Xu et al., 2021), colon adenocarcinoma (COAD), rectum adenocarcinoma (READ), and stomach adenocarcinoma (STAD) (Xu et al., 2021). However, the gene expression of ANGPTL8 was decreased in CHOL, BRCA, LUAD, LUSC, UCEC, BLCA KICH, GBM, and UCEC (Table 1) (Xu et al., 2021; Iqbal et al., 2023). It has also been found that ANGPTL8 levels are elevated in pancreatic adenocarcinoma (PAAD)-associated diabetes patients (Susanto et al., 2016). Furthermore, ANGPTL8 levels were associated with poor prognosis in KIRC, UCEC, and LUSC patients (Xu et al., 2021) but with longer overall survival or disease-free survival time in CHOL, GBM, sarcoma (SARC), uveal melanoma (UVM), BLCA, BRCA, pheochromocytoma and paraganglioma (PCPG), thymoma (THYM), uterine carcinosarcoma (UCS) patients (Xu et al., 2021; Iqbal et al., 2023) and PAAD (Taherkhani et al., 2020) (Table 1). An integrated computational analysis revealed that 4 SNPs in ANGPTL8 were predicted to be involved in large intestine, breast, and liver cancer (Iqbal et al., 2023).

**TABLE 1:** ANGPTL8 gene expression and the association with prognosis in different cancers.

Cancer	ANGPTL8 gene expression	High ANGPTL8 expression with prognosis	Reference
KIRC/ccRcc, COAD	↑	Poor	Xu et al. (2021), Matsukawa et al. (2023)
HCC	↑	Poor	Wang et al. (2018), Monzavi et al. (2019)
		Favorable	
GBM, CHOL, BRCA, BLCA	↓	Favorable	Xu et al. (2021), Iqbal et al. (2023)
UCEC, LUSC	↓	Poor	Xu et al. (2021)
STAD, READ	↑	Not reported	Xu et al. (2021)
LUAD, KICH	↓	Not reported	Xu et al. (2021)
THYM, UCS, UVM, SARC, PCPG, PAAD	Not reported	Favorable	Xu et al. (2021), Taherkhani et al. (2020)

Abbreviations: bladder urothelial carcinoma (BLCA), breast invasive carcinoma (BRCA), clear cell renal cell carcinoma (ccRcc), cholangiocarcinoma (CHOL), colon adenocarcinoma (COAD), glioblastoma (GBM), hepatocellular carcinoma (HCC), kidney chromophobe (KICH), kidney renal clear cell carcinoma (KIRC), lung adenocarcinoma (LUAD), lung squamous cell carcinoma (LUSC), pancreatic adenocarcinoma (PAAD), pheochromocytoma and paraganglioma (PCPG), rectum adenocarcinoma (READ), sarcoma (SARC), stomach adenocarcinoma (STAD), thymoma (THYM), uterine corpus endometrial carcinoma (UCEC), uterine carcinosarcoma (UCS), uveal melanoma (UVM).

The potential mechanism of ANGPTL8 in cancers has not yet reached a consensus. ANGPTL8 may be induced by STAT3 signalling (Matsukawa et al., 2023), FFAs (Liu et al., 2018; Zhang et al., 2023) or other proinflammatory cytokines, such as TNF-α (Zhang et al., 2017) and TGFβ1, in the tumour microenvironment, and it may affect the differentiation and development of tumours through the following pathways. First, ANGPTL8 may inhibit cell proliferation and promote apoptosis by affecting the Wnt signalling pathway, increasing the expression of the tumour suppressor gene WIF1 and downregulating the antiapoptotic gene Bcl2 (Monzavi et al., 2019; Taherkhani et al., 2020). Second, the interaction of the C-terminus of ANGPTL8 with sterol regulatory element binding protein 1 (SREBP1) inhibits protein kinase activated by adenosine 5′-monophosphate (AMP)-activated protein kinases (AMPK) (Navaeian et al., 2021; Lee et al., 2015); therefore, they increase lipogenesis and tumour growth and enhance tumour progression (Navaeian et al., 2021). Third, as mentioned earlier, ANGPTL8 negatively regulates inflammation and autophagy by inhibiting NF-κB activation, which was confirmed to be crucial to the progression of cancer (Perkins, 2012; DiDonato et al., 2012). Fourth, the ANGPTL protein family is also associated with the regulation of stem cell activity (Endo, 2019; Zheng et al., 2012; Santulli, 2014). Therefore, ANGPTL8 may affect cell proliferation by modulating stem cell activity. Fifth, ANGPTL8 was demonstrated to engage with 24 amino acid residues within RASSF5 proteins. These proteins play vital roles in various cellular processes, including the regulation of the cell cycle, cell proliferation, and differentiation (Iqbal et al., 2023). Furthermore, the deactivation of RASSF5 has been associated with the initiation of oncogenic processes (Iqbal et al., 2023). In summary, the potential role of ANGPTL8 in tumours may be complicated and involve multiple pathways.

## 4 ANGPTL8 and the circulatory system

ANGPTL8, a factor involved in regulating lipid metabolism, serves as a potential biomarker for metabolic diseases, which are closely associated with cardiovascular diseases. Recently, it was also reported to be involved in the pathophysiological processes of circulatory system-related diseases as an inflammatory factor.

Atherosclerosis is a major cause of cardiovascular disease (Brandsma et al., 2019). Atherosclerosis has been extensively linked to lipid infiltration and inflammation in numerous investigations (Libby, 2021). Notably, studies have revealed elevated serum ANGPTL8 levels in coronary heart disease patients relative to their healthy counterparts, and a positive correlation was observed between ANGPTL8 levels and disease severity (Jiao et al., 2018). This connection has spurred researchers to investigate the role of ANGPTL8 in atherosclerosis progression. Findings have indicated an upregulation of ANGPTL8 expression in atherosclerotic mice, and the improvement of atherosclerosis was observed after knocking out ANGPTL8 (Zheng et al., 2018). Jiao et al. (2018) investigated the location of ANGPTL8 in atherosclerosis using immunofluorescence and found that ANGPTL8 is expressed in macrophages within atherosclerotic lesions. Overexpression of ANGPTL8 in macrophages led to foam cell formation and extensive cholesterol deposition, promoting the development of atherosclerosis (Jiao et al., 2020); this suggests that the influence of ANGPTL8 on blood vessels is not solely through the regulation of triglyceride levels. Additionally, studies have shown that inhibiting ANGPTL8 using second-generation 2′-O-methoxyethyl (ASO) can prevent cardiovascular disease (Morelli et al., 2020). However, contrary to the mainstream notion, a prospective study of 553 coronary heart disease patients followed for 8 years indicated that elevated plasma levels of ANGPTL8 have a protective effect on the heart and are associated with a lower risk of cardiovascular events and mortality (Leiherer et al., 2020). The specific mechanisms require further investigation.

### 4.1 Hypertension

Circulating ANGPTL8 levels were significantly higher in hypertensive patients, as well as in mice and rats (Abu-Farha et al., 2018; Xu et al., 2023; Jiao et al., 2023). It is generally believed that this association is due to its role in LPL inhibition. Jiao et al. (2023) reported that targeted removal of ANGPTL8 in vascular smooth muscle cells (VSMCs) mitigated AngII-induced hypertension and hypertensive cardiovascular remodelling. They demonstrated that ANGPTL8 regulates hypertension and that arterial remodelling involves accelerated constriction, proliferation, and migration of VSMCs through the promotion of PI3K-AkT pathway activation.

### 4.2 Cardiomyopathy

Serum ANGPTL8 levels were elevated in patients with hypertensive left ventricular hypertrophy (Hu et al., 2022). ANGPTL8, as an endocrine protein, is not expressed in cardiomyocytes but is mainly expressed in primary mouse liver cells and HepG2 cells. In mice with angiotensin II (Ang II)-induced cardiac hypertrophy or transverse aorta constriction (TAC)-induced cardiac hypertrophy, serum ANGPTL8 levels were also significantly increased (Su et al., 2021). Importantly, these mice do not exhibit lipid metabolism disorders, suggesting that the impact of ANGPTL8 on the heart is not mediated through the regulation of blood lipids but may involve direct effects on cardiomyocytes. Hu et al. (2022) found that ANGPTL8 can bind to LILRB3 and block downstream signalling pathways, such as those of AKT and glycogen synthase kinase-3β (GSK3β), thereby improving pathological cardiac hypertrophy. ANGPTL8 deficiency accelerates Ang II-induced cardiac hypertrophy and fibrosis (Su et al., 2021). Wang et al. (2022) also discovered a negative correlation between ANGPTL8 and left ventricular mass index (LVMI), indicating a protective effect of ANGPTL8 on cardiac remodelling. Thus, cardiac hypertrophy may upregulate the expression of ANGPTL8 through certain mechanisms, and circulating ANGPTL8 can directly act on cardiomyocytes to exert anti-ventricular remodelling effects.

## 5 ANGPTL8 and ectopic fat deposition

Mesenchymal stem cells (MSCs) are multipotent stem cells with self-renewal and multilineage differentiation potential, including differentiation into adipocytes (Fu et al., 2019). When body fat exceeds the normal threshold, MSCs differentiate into adipocytes to meet the body’s energy storage needs. Tang et al. (2022) demonstrated that ANGPTL8 promotes adipogenic differentiation of MSCs in liver, kidney, and heart tissues by inhibiting the Wnt/β-catenin signalling pathway. This upregulates the expression of peroxisome proliferator-activated receptor γ (PPARγ) and c/EBPα and leads to bone marrow fat deposition (Sun et al., 2017). Ectopic bone marrow fat deposition is closely related to chronic metabolic diseases, such as T2DM, NAFLD, and obesity (Tang et al., 2022).

## 6 Conclusion

In summary, ANGPTL8 is primarily secreted by the liver and adipocytes. Previous studies have mainly focused on improving lipid profiles in individuals with metabolic risk factors by creating antibodies targeting ANGPTL3, ANGPTL4, and ANGPTL8. In recent years, it has been discovered that ANGPTL8 exerts different biological effects in different locations (including within and outside blood vessels and target cells) and participates in various pathological processes, such as inflammation, tumour processes, ventricular remodelling, and ectopic fat deposition. These findings suggest that ANGPTL8 may be involved in metabolism or inflammation across multiple organ systems. Further research is needed to investigate the effects of ANGPTL8 in various disease states, its cellular and extracellular actions, and its precise molecular mechanisms.
